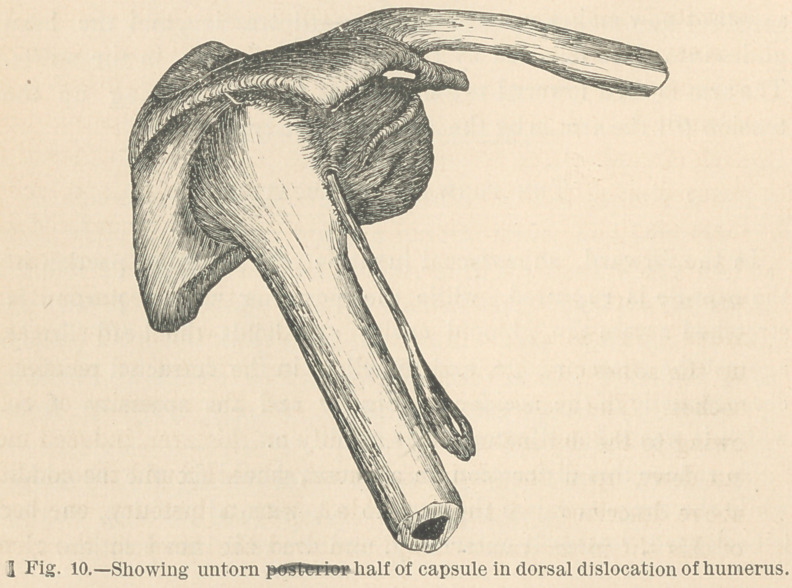# The Philosophy of Manipulation in the Reduction of Hip and Shoulder Dislocations

**Published:** 1884-05

**Authors:** Moses Gunn

**Affiliations:** Prof. of Surgery, Rush Medical College


CHICAGO MEDICAL

JOURNAL & EXAMINER.

VOL. XLVIII.—MAY, 1884.—NO. 5.

### Original Communications.

ARTICLE I.

### The Philosophy of Manipulation in the Reduction of Hip and Shoulder Dislocations. (From the Transactions of the American Surgical Association. Annual Session of April 30, May 1, 2, and 3, 1884, Washington, D. C.) By Moses Gunn, M.D., LL.D., Prof. of Surgery, Rush Medical College.

*Mr. President and Fellows:*

It is now a third of a century since Dr. Reid, of Rochester, N. Y., proposed a definite method of manipulation for the reduction of the upward and backward dislocation of the femur.

By his method he sought to overcome the resistance which certain structures, in his estimation, offered to the reduction; and, although he was in error as to the particular structures offering such resistance, he is entitled to the credit of original and thoughtful investigation of the subject, and the proposal of a definite method of manipulation. Wonder is also aroused that, while his investigations carried him so near the exact truth, he failed to recognize and grasp it. On this point I shall dwell at a more advanced stage of this paper.

After the publication of Dr. Reid's paper in the "Transactions of the Medical Society of the State of New York, 1852," there were numerous contemporaries who failed to see anything new or original in his method, and they called attention to the practice of Dr. Nathan Smith, of New Haven, who was in the habit of reducing dislocations without extension and counter-extension, simply by certain movements, and without the exercise of great power. Other authorities were also cited to prove that the reduction of dislocations by manipulation was not new, the critics overlooking the fact that Dr. Reid's method was a definite plan to overcome the resistance offered by certain *recognized* agents, and not a mere manipulation having only a general result in view.

Exactly what Dr. Smith's method was seemed not to be known, even by those who had sat at his feet. That, however, he reduced dislocations by manipulations of some sort, was satisfactorily established.

Since the publication of Dr. Reid's paper, and the discussions which it occasioned, the practice of manipulation in the reduction of dislocations, not merely of the dorsal variety in the hip, but of all dislocations of both hip and shoulder, has become frequent, though by no means general. The manipulations, too, are often practiced somewhat vaguely, without a clear conception of the nature of the difficulties to be overcome.

The purpose of this paper is to discuss these difficulties and the methods best calculated to prevail over them, and if in so doing I shall dwell somewhat at length upon my own thought, investigation, and teaching, in reference to this subject, during a period dating from the publication of Dr. Reid's paper, I trust that your indulgence will acquit me of an undue display of egotism.

Previous to the publication of Dr. Reid's article, the opinion was, probably, universal, that muscular action or spasm holding the dislocated member in its abnormal position, constituted the resistance to our efforts, and the obstacle to be overcome in reducing hip and shoulder dislocations. Hence the continuously prolonged extension, for the purpose of tiring out the muscles. Hence, also, in supplementing the effect of such efforts to overcome muscular action, bloodletting, tartrate of antimony, and various other means of diminishing muscular energy, were often invoked.

Dr. Reid, however, recognizing the fact that muscular action was easily enough overcome in cases of fracture of the neck and trochanteric portion of the femur, and that muscular action could not be present in his experiments on the cadaver, sought for the resistance in the physical tenacity of the muscular structures about the joint. In this idea of Dr. Reid, we have a recognition of the physics of the dislocated condition, disassociated from physiological activity. I have already given expression to wonder that Dr. Reid failed to grasp the whole of this physical potency, overlooking its major part, when in one of his experiments it was thrust so prominently before him. In detailing this experiment, which was upon the cadaver, he says:

" * * * * although all the muscles about the joint were separated from each other, were loose, without vitality, and almost in a state of decomposition, yet it was with great difficulty that we could bring down the head into its socket; and when we did so, we carried away a part of the capsular ligament."

The significance of the fact stated in this last clause was lost to the observer, as it was, also, to me in the first reading of the article; for not until a reperusal of the paper ~~seven~~ years subsequent to the first reading, when I had in the meantime fully elaborated the views which I shall advance in this paper, did its significance challenge my attention. In his efforts at reduction he *carried away a part of the capsular ligament;* the reduction was not effected till the *ligament was sacrificed:* and yet he saw no obstacle to the reduction except in the *half rotten muscles* which, he says, "constitute the real and only impediment."

Stimulated by Dr. Reid's article and by the demands of the surgical chair which, in consequence of my then youthful age, I was earnestly striving to fill, in the equally youthful University of Michigan, I began a careful investigation of the subject, and at an early period in my experiments, I recognized in the untorn portion of the capsular ligament the structure which held the head of the dislocated femur or humerus more or less firmly locked in its luxated position, and thus constituted the great and principal, if not the only, impediment to be overcome in reducing a hip or shoulder dislocation. In a paper which I read before the Detroit Medical Society, and which was published in the *Peninsular Journal of Medicine* in September, 1853, I used the following language:

"What structure stood between effort and success? I answer, *the untorn portion of the capsular ligament.* * * * * Extension and counter-extension by the pulley or Jarvis's apparatus in the usual direction, succeeds only by lacerating much more extensively, if not by tearing the ligament completely asunder, before the head will ride over the edge of the cavity. The principle, then, I would seek to establish is this: *that in luxations of the hip and shoulder, the untorn portion of the capsular ligament, by binding down the head of the dislocated bone, prevents its ready return over the edge of the cavity to its place in the socket; and that this return can be easily effected by putting the limb in such a position as will effectually approximate the two points of attachment of that portion of the ligament which remains untorn.*"

In an extension and republication of this paper, six years later, I added to the above principle the following:

"For the easy reduction of a dislocation, the dislocated limb should be placed in exactly that position which characterized it at the moment of escape of the joint end from its normal position in the joint."

These two principles constitute the key to the whole subject of manipulation in the reduction of dislocations; and the first, viz. that which refers to the untorn portion of the ligamentous capsule is fully vindicated by Professor Bigelow in a work on the Hip, published in 1869. This distinguished surgeon and teacher, however, as is well known, dignifies the reinforcing fibers of the ilio-femoral ligament by giving to it (the ilio-femoral) distinct individuality, and applies to it the term "Y ligament." The correctness or propriety of this particular anatomical individualization need not be dwelt upon in this connection. To that part of the anterior portion of the capsule which is covered and strengthened by these fibers which blend with the capsular structure, he limits the potency which gives the characteristic deformity to the various dislocations of the hip, and which, he thinks, opposes our efforts in reduction by the old method of extension and counter-extension.

This portion of the capsule is, manifestly, much the strongest, and is probably rarely torn asunder in any of the four classical dislocations, except the thyroid, in which it is, probably always, completely ruptured, as I shall have occasion to demonstrate in the course of the present paper. Its entire want of influence in the dorsal variety of dislocation I shall also be able to show by exhibition of a dissection of the parts.

This is the portion of the capsule which Professor Bigelow claims to remain untorn in all hip dislocations; and his manipulations are so directed as to effect its relaxation, thus coinciding with the first of the two general principles above named. These principles I shall endeavor to elaborate, illustrate, and apply.

Before, however, proceeding to this branch of my subject, I desire to direct attention to another structure which plays an assisting rôle in holding the head of the femur down outside the ridge of the acetabulum in the dorsal dislocation. If, in an intact state of the muscles and the external portion of the fascia lata, the capsular and round ligaments be completely divided, and the head of the femur be luxated upon the dorsum of the ilium, it will be found that the characteristic deformity of direction in the limb will be wanting, *i. e.* the limb will be parallel with its fellow, on a line with the trunk lacking the inversion and adduction, but will be shortened the usual extent. If now the limb be placed in the position characteristic of dorsal luxation in the living subject, and the reduction be attempted by the old method of extension and counter-extension, it will be found that the head is still held down firmly in its hooked position outside of the ridge of the acetabulum. It is thus held by the fascia lata, which in this position of the limb describes the outermost curve, and consequently is put upon the stretch and holds the whole trochanteric end of the bone pressed firmly inwards. But by taking care to rotate the limb well inwards, so as to avoid the pressure of the fascia lata upon the great trochanter, a moderate amount of extension will draw the head into the socket. Hence, in all manipulations for the reduction of the dorsal or ischiatic dislocations, the limb should be kept well rotated inwardly.

Let us now first proceed to a consideration of the last of the above-named principles, viz.: that which calls for the putting of the limb in the position which characterized it at the moment of escape. It is often impossible to obtain direct and positive evidence on this point, either from the patient himself or from the witnesses of the accident; but occasionally we are more fortunate, and the evidence of these fortunate occasions is of great value in estimating the probabilities in cases when positive evidence is wanting. It is rare that dislocation of a healthy and perfect shoulder-joint will occur except in a distorted position of the humerus at the time of the escape of the head from the glenoid cavity, and it is not probable that a dislocation of a healthy and perfect hip-joint can ever occur except in a very distorted position of the femur at the moment of escape. In the hip-joint of average perfection, it is tolerably certain that either pretty extreme adduction, abduction, internal rotation, or external rotation must be present before a dislocation can take place. I lay down the rule with full confidence in its correctness, that so long as the femora are parallel with one another, on a line with the longitude of the trunk, and midway between eversion and inversion, violence may effect a fracture, but not an uncomplicated dislocation. Before a dislocation can occur, an extreme degree of distortion in one, and sometimes two or more of the above-named directions, must be present. For instance, in the dorsal dislocation the femur must be adducted, flexed, and internally rotated before escape is possible. In the thyroid form of the accident, extreme abduction and moderate eversion must preëxist. In the backward form, flexion at about a right angle with the body, associated with extreme adduction, or, if flexion and adduction are not present, extreme internal rotation, must characterize the position of the femur at the instant of the escape. In the pubic luxation, extreme eversion, with probable moderate abduction, antedates the escape of the head from the socket.

Illustrative evidence in support of these assumptions is frequently at hand in the manner in which the accident occurred. Thus, the man who in falling from a height struck upon the foot in an adducted position of the limb, by which the limb was still more powerfully adducted, sustained a dorsal dislocation.

A mechanic, in a sitting position before a car platform, had the car suddenly moved toward him, striking the knee and tilting the pelvis so as to produce relatively a strongly adducted position, added to the right-angled flexion of the limb incident to the sitting position, and he sustained a backward dislocation.

A lad fell before a low-wheeled stone-truck, which passed over him, and to which he was forced to enact the part of roller, whereby, undoubtedly by pressure on the trochanter, he had the limb forced into an extreme position of external rotation, and he was picked up with pubic dislocation.

A man slipped outward on one foot, coming down with the leg forced outwards to a condition of extreme abduction, and he sustained a thryoid dislocation. Such examples, quadrating accurately with mechanical principles, strongly attest the correctness of the positions above assumed.

In the shoulder joint, owing to the shallowness and relatively small size of the glenoid cavity, this rule of distortion antedating dislocation does not necessarily obtain, but we know that it is, frequently, not only present, but is, also, the proximate cause of the accident. For instance, direct violence, operating from a direction outward upward upon the head of the humerus, may produce the axillary dislocation; and a variation in the direction of the force, from outwards and forwards, or from outwards and backwards, may produce either the dorsal or thoracic displacement; but more frequently we are furnished with demonstrative evidence of the fact that extreme distortion is one of the actual and proximate causes of the luxation.

Thus, the axillary dislocation is generally caused by the suddenly enforced position of the arm upwards by the side of the head, while the forward luxation is usually produced by falling backward, the arm being thrown backward to break the fall; and the backward displacement is effected in a similar manner, by throwing the arm forward to break a plunging fall in that direction.

Now, it is certain that in the position in which the limb was at the instant of escape, that escape was entirely consistent with the existing condition of all the structures about the joint; and, in the same position, the joint end of the dislocated bone ought to travel back with equal facility, by a simple reversal in the direction of the force.

The exception to this position will be found in rare cases, where by a simple splitting of the fibers of the capsule, a button-hole form of laceration occurs which grasps the neck of the completely escaped head. This condition, when it is encountered, requires something more than manipulation; more extensive rupture must be effected.

But, in ordinary cases, it is evident that the position which facilitates or permits the escape, will also be favorable to the return of the escaped head. It seems needless to appeal to mechanical principles in justification of this position. Alter the position, and relations are changed; impediments and obstructions are found in the desired route of return, and reduction becomes difficult or impossible without undue force, and injury to the opposing structures. Restore the position and relation of parts, and the reduction becomes practicable with the exercise of moderate force. The position of the limb at the instant of escape, when not established by direct evidence, can be assumed with tolerable accuracy by the application of the general principles which we have considered above.

I think, therefore, that in reference to position, I may offer the general rule: *That for the easy reduction of a dislocated hip or shoulder, the limb should be placed in, as nearly as possible, the same position as that which most frequently characterizes it at the instant of escape.*

It now remains to consider the principle first named, viz.: *That the reduction of hip and shoulder dislocations can generally be easily effected by putting the limb in such a position as will effectually approximate the two points of attachment of that portion of the ligament which remains untorn.*

But how are we to ascertain which portion remains untorn?

It is obvious that in primary dislocations, *i. e.*, when the dislocated head remains in the locality where it was first thrown, the portion of the ligament on the side toward which the head has been transplanted must be so extensively torn as to permit the escape of the head and its progress to its dislocated position. It is also equally obvious that the opposite portion is not necessarily torn, and when we consider that the position of the limb at the moment of escape is such as to relax this portion of the ligament, we can safely assume its integrity. Take, for example:

### THE DORSAL LUXATION OF THE HIP.

The superior and posterior portion of the ligament must be torn, or there could be no dislocation in this direction, while, from the position of the limb at the instant of luxation, the anterior and inferior portion, being relaxed, remains untorn. Now, if we adduct, flex, and inwardly rotate the limb, we approximate the points of attachment of the untorn portion, and thus effectually relax it. If we now apply force in the reverse direction to that which produced the luxation, we shall be able to replace the dislocated head by the exercise of that amount of force which was necessary to cause the accident, minus the amount of force required to rupture the ligaments and tissues which are torn asunder.

The condition of the ligament in this luxation is illustrated in Figs. 1 and 2, Fig. 1 being an anterior, and Fig. 2 a posterior view of the same specimen.

Fig. 1.—Anterior view, showing tense condition of anterior and inferior portion of capsule, and the loose state of the ilio-femoral portion in the dorsal dislocation.

Fig. 2.—Posterior view of same specimen, showing the tense state of the anterior and inferior untorn portion of capsular ligament.

These figures—as is the case in all my illustrations—are made from a dissection of the parts, which dissection I also herewith exhibit. It is seen that the anterior and inferior portion of the ligamentous capsule is untorn, tense, and holds the dislocated head firmly hooked outside the dorsal portion of the rim of the acetabulum, while that portion of the capsule between the anterior inferior spinous process of the ilium and the anterior intertrochanteric line of the femur, which is reinforced and strengthened by the ilio-femoral fibers, is quite loose, owing to the aproximation of these two points, in the shortened, adducted, and internally rotated state of the limb which characterizes this form of dislocation. Thus, this ilio-femoral portion of the capsule, in the dorsal luxation, is entirely without influence, either in determining the deformity or in opposing our efforts at reduction. It is entirely to the anterior and inferior portion of the capsule that these influences are due.

If we now flex, adduct, and inwardly rotate to a still greater degree, we shall loosen the anterior and inferior tense untorn portion which is holding the head hooked outside the acetabular ridge, and then by a moderate amount of force we may draw the head into the socket. This is most conveniently accomplished by putting the patient on the floor on his back ; an assistant fixes the pelvis ; the surgeon grasps the limb, flexes and adducts it till it crosses the limb of the opposite side at a point as high as the union of the upper with the lower two-thirds of the femur ; now rotating the limb inwardly, he will be able to lift the head into place by a moderate effort.

### THE BACKWARD DISLOCATION OF THE HIP.

In this variety, whether produced by force operating on the great trochanter rotating the femur forcibly inwards, or by force applied to the lower end of the bone while it is flexed to a right angle with the trunk, it is evident that the posterior portion of the capsular ligament is ruptured, and it is nearly as certain that the anterior portion remains untorn. This anterior unruptured half is drawn tensely across the acetabulum and holds the head hooked behind the posterior portion of the acetabular wall. This is shown in Fig. 3.

Fig. 3.—Showing tense condition of anterior half of capsular ligament in backward dislocation.

Extreme internal rotation, or flexion to a right angle with the trunk and adduction, either, will relax the anterior untorn portion of the ligament, and allow an easy transit of the head over the edge of the acetabulum.

Perhaps the most practical method of applying to this form of dislocation the principles which I have advocated, will be found in placing the patient on the floor on his back, in the same position recommended in the dorsal luxation. An assistant fixes the pelvis while the surgeon flexes the thigh at a right angle with the trunk, and the leg upon the thigh; he then adducts, rotates inwardly, and draws the limb forwards in the direction of extreme adduction, thus lifting the head directly into the socket.

### THE THYROID DISLOCATION.

In the dislocation downwards and forwards over the thyroid foramen, the anterior and inferior portion of the capsular ligament must be torn asunder for the escape of the head; while from the extremely abducted state of the limb at the moment of the accident, the superior and posterior portion must be relaxed, and thus escape laceration.

Fig. 4.—Showing the tense, untorn, upward and backward portion of capsular ligament in thyroid dislocation.

Fig. 4 illustrates this luxation and the condition of the ligament. It is seen that while the head of the femur occupies a position over the thyroid foramen, and while the characteristic deformity of direction in the limb is present, viz.: a moderately flexed and slightly abducted position, the superior and posterior untorn portion of the ligament is tense and holds the limb in its state of slight abduction. The flexed position of the limb is due mainly to the necessarily tense condition of the psoas magnus and iliacus muscles.

The characteristic position of the limb in this dislocation is inconsistent with the integrity of the ilio-femoral portion of the capsular ligament. The greatly increased distance between the anterior inferior spinous process of the ilium and the anterior inter-trochanteric line of the femur cannot be accommodated by anything less than the rupture of this portion of the ligament. The head of the femur *can* be placed over the thyroid foramen in the intact state of this portion of the ligament; but in order to accomplish this, the femur must be flexed to a right angle with the longitude of the trunk. This is illustrated in Fig. 5.

Fig. 5.—Showing what would be the degree of flexion in thyroid dislocation if the ilio-femoral portion of capsule remained untorn.[1]

An examination of this figure, or of the specimen which I herewith exhibit, will fully warrant the positive statement, that in the downward and forward luxation, if the limb is found in the position generally characteristic of this form of the accident, the only untorn part of the capsule will be the upward and backward portion, as is illustrated in figure 4.

To reduce this dislocation, the reducing force should be applied in the usual way to the inner aspect of the upper part of the thigh at its junction with the perinæum, with the intention of lifting the head directly into the socket; but instead of adducting the limb, as is the usual practice, free abduction should be made, thus fulfilling both of the general principles which I have laid down. With these manipulations, there is nothing in the way of an easy return of the head to the socket.

### THE FORWARD DISLOCATION.

The forward or pubic dislocation is the most complicated, and consequently the most interesting of the four principal forms of hip luxation. It is probably the result of force applied to the trochanter major in such a manner as to rotate the limb forcibly outwards, and in this position forcing the head forwards. As the head escapes from the socket and travels forward to its position in front of the body of the pubis, it is certain that the anterior portion of the capsule is ruptured, and it is nearly equally certain that the rupture occurs at the weakest part of this anterior portion. The weakest part will be found at the internal extremity of the ligament at its attachment to the rim of the acetabulum, where it is not reinforced by the ilio-femoral fibers. Through this opening the head escapes and rests in front of the body of the pubis, the posterior surface of the neck resting on the edge of the acetabulum, and the posterior border of the great trochanter settling somewhat into the socket. The portion of the capsule which remains untorn is the whole of the posterior half, and that part of the anterior half covered and strengthened by the reinforcing ilio-femoral fibers. The posterior half is forced down into the acetabulum by the trochanter major, which encroaches upon that cavity. This is shown in Fig. 6, which is an external view of this dislocation :

Fig. 6.—External view of pubic luxation, showing the posterior border of the great trochanter setted into the acetabulum, pressing before it the posterior untorn half of capsule.

Thus pressed into the cavity, this posterior portion of the capsule is moderately tense, but it does exert much influence on this dislocation in any way. On the contrary, the ilio-femoral portion of the capsular ligament in front having continuity of structure with the posterior untorn portion from below the cervix, holds the dislocated head in its luxated position. In this dislocation, the ilio-femoral portion of the capsular ligament, by its continuity with the inferior border of the posterior untorn portion, possesses the potency which Professor Bigelow claims for it in all dislocations. Its position and form, in this luxation, are illustrated in Fig. 7:

Fig. 7.—Anterior view of pubic luxation, showing continuity of tissue between the ilio-femoral and inferior border of posterior half of capsular ligament in pubic dislocation.

It is seen by consulting this illustration, and by examining the preparation from which it is taken, that the influence exerted by this part of the capsular ligament depends largely, if not entirely, upon its continuity with the inferior border of the posterior untorn portion. With this continuity, it has pelvic attachment at each end, while the central portion lies over the cervix and holds it in its dislocated position. To elude the grasp of this untorn portion of the ligament, we have simply to reverse the direction and successive order of the dislocating force. That force produced, first, extreme external rotation; second, pressure inwards and forwards. Now, to reverse this force, the limb should be drawn backwards and outwards, without altering its general position as regards rotation and direction; then rotate inwards, thus reversing the force both in direction and order.

Practically, the patient should be placed upon the edge of a firm table—a square piano is better on account of its height—with the dislocated limb hanging over the border. An assistant fixes the pelvis while the surgeon, kneeling on the floor, grasps the limb and flexes the leg upon the thigh to obtain more perfect control of the parts. He then draws the limb backwards and outwards, and supplements these efforts by internal rotation, when the head slips into the socket.

### DISLOCATIONS OF THE SHOULDER.

Owing to the comparatively small size and shallowness of the glenoid cavity, relatively considered with the head of the humerus, and owing, also, to the looseness of the joint, whereby extreme latitude of motion is provided for, dislocations of the shoulder occur from the operation of comparatively slight violence, and they are reduced with corresponding facility; but the same rôle is played by the capsular ligament here as in the hip-joint, and the same mechanical principles are involved in efforts at reduction.

### THE AXILLARY DISLOCATION.

In this dislocation, the inferior portion of the capsule is lacerated, while the superior remains untorn, tense, and—during the first few hours, and sometimes days, until the head works well up into the axillary space—acts as a check-strap upon the humerus, keeping the elbow away from the trunk. This condition of the ligament is shown in Fig. 8, and also in the specimen from which the figure is made, and which I also herewith exhibit. If the humerus be carried up by the side of the head, the untorn portion of the ligament becomes entirely lax, and moderate effort will now draw the head upward into the cavity.

Fig. 8.—Showing untorn upper part of capsule in axillary dislocation of humerus.

For the reduction of this dislocation it is convenient to have the patient sit upon the floor. The arm is then raised to an angle of 45 degrees from the horizontal, and intrusted to an assistant, while the surgeon places his hands on the shoulder with the tips of the fingers in the axilla, resting on the dislocated head. The assistant now makes upward and outward traction, and the head glides into place followed by the surgeon's fingers in the axilla. The arm is then lowered to the pendent position, keeping up the tension till the arm is by the side of the body.

### THE FORWARD DISLOCATION.

In the forward, subcoracoid luxation, the anterior portion of the capsule is ruptured, while the posterior untorn portion is stretched across the glenoid cavity and holds the head firmly against the anterior edge, and the elbow in the retracted position characteristic of the accident. Fig. 9 and the specimen from which it was taken illustrate this condition.

To reduce this dislocation an assistant should fix the shoulder while the surgeon raises the arm to a horizontal position, carries it backward, rotates it externally, and draws it into position.

Fig. 9.—Showing untorn posterior half of capsule in sub-coracoid dislocation of humerus.

THE DORSAL DISLOCATION.

In this accident we have the same condition of the parts as in the forward luxation, only reversed in antero-posterior direction.

anterior | Fig. 10.—Showing untorn ~~posterior~~ half of capsule in dorsal dislocation of humerus.

Fig. 10 and the original specimen which is here exhibited fully demonstrate this lesion. It is seen that the head rests on the dorsum of the scapula, while the vacated glenoid cavity is covered by the anterior untorn half of the capsular ligament, which is stretched across the articular surface, holding the head snugly against the posterior edge of the fossa, and by its inferior fibers causing the advanced position of the lower end of the humerus, which is so characteristic of the accident. Internal rotation relaxes this untorn portion of the ligament, as does also a still more advanced position of the elbow with the humerus elevated to a horizontal position.

For a reduction of this luxation the shoulder should be properly fixed by an assistant, while the surgeon seizes the arm by the elbow and forearm, raises it to a horizontal position, carries it to the front, rotates inwardly and draws it into place.

### ANOMALOUS DISLOCATIONS.

When we consider the discrepancy in the size of the humeral head and the glenoid cavity, and observe the amplitude of the capsular ligament which is necessary for the extreme latitude of motion enjoyed by the superior extremity, we notice that the marked fullness of the capsule is gathered up, at its internal extremity, to the size of the rim of the glenoid cavity, thus giving it a somewhat ruffled form. Such a construction, when perhaps it is unusually marked, may permit a splitting of the fibers, giving a button-hole form to the laceration. The escaped head, under such circumstances, would be firmly grasped by the edges of this fissured opening in the capsule, in such a manner as to foil all mere manipulatory efforts. I have three times encountered what I have considered to be this state of the parts. In one case it was my fortune to be able to demonstrate the correctness of these views. It was an old forward dislocation, when, after breaking up the adhesions, I was unable to cause the head to reënter the socket. The uselessness of the arm and the necessity of relief, owing to the dependence of a family on this arm, induced me to cut down upon the dislocated head, when I found the condition above described. I freely divided, with a bistoury, one border of this slit in the capsule, and replaced the head in the glenoid fossa. This experience was before the era of antiseptic precaution, and although a prolonged suppurative history followed, a final satisfactory recovery was realized.

The other two cases were recent axillary luxations in which no manipulatory effort was sufficient to alter the relation of the displaced head to the socket. Free rotation, backwards and forwards, through nearly all the three hundred and sixty degrees, failed to enlarge the opening sufficiently to permit reduction. Resort was then had to the compound pulley, and extension carried to the ultimate verge of temerity produced signs of laceration of ligamentous structures, but no snap of reduction. Extension was discontinued, and then simple manipulation reduced the luxation at once.

## Figures and Tables

**Fig. 1. f1:**
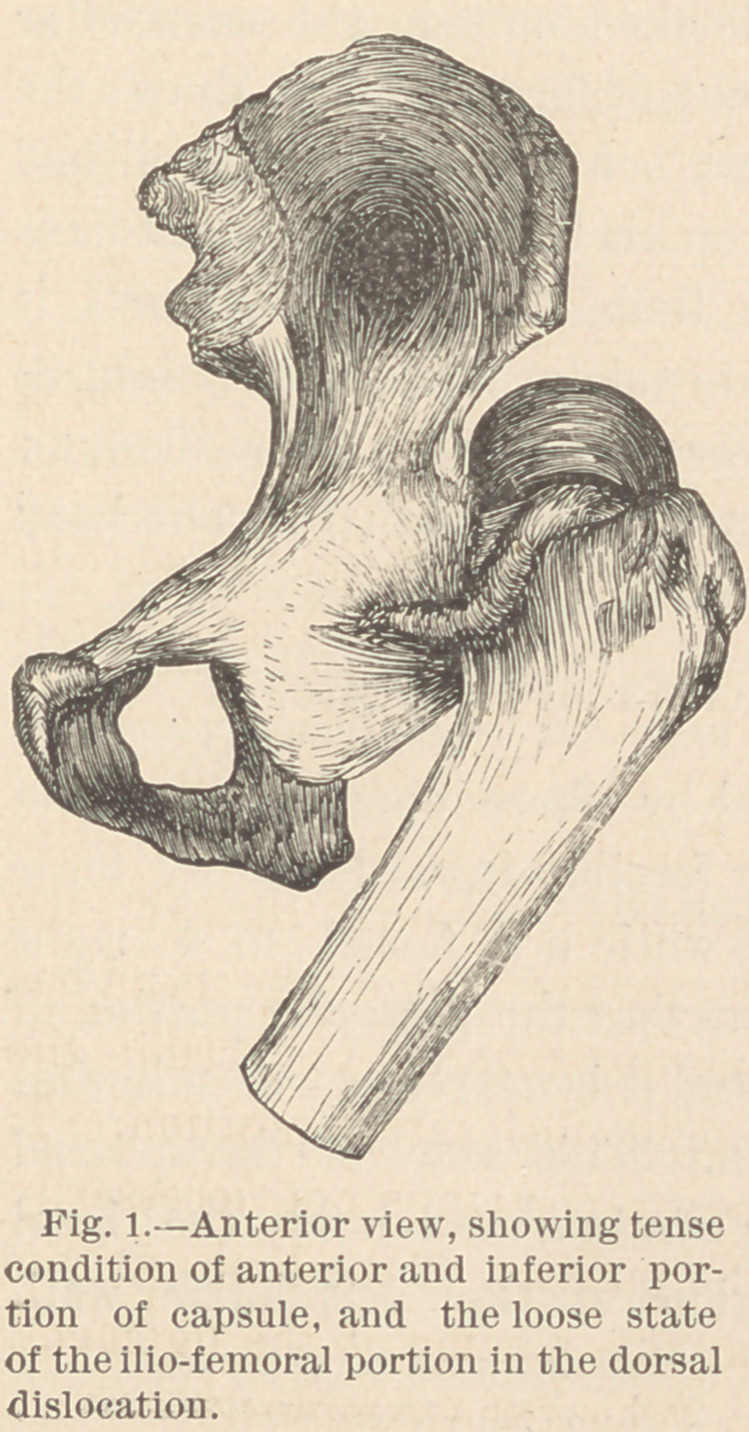


**Fig. 2. f2:**
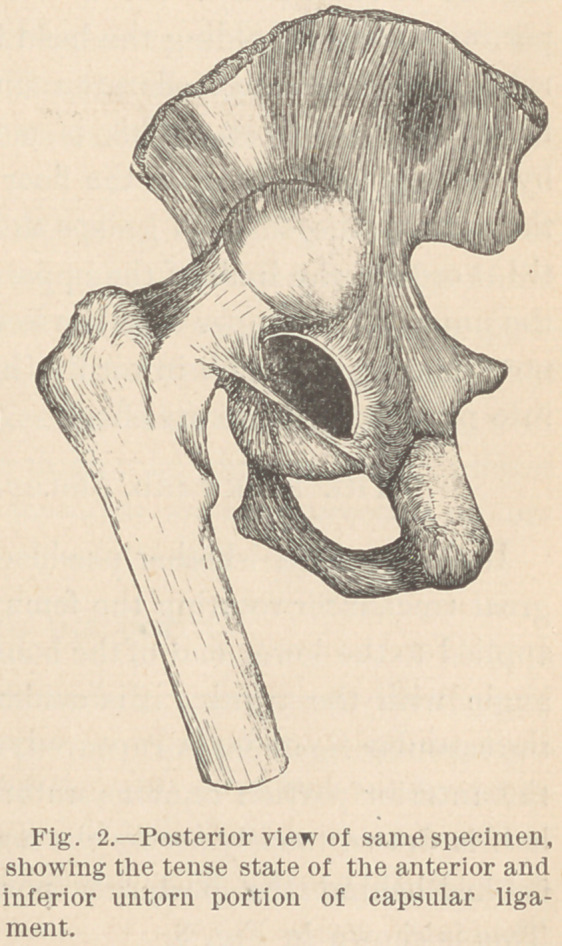


**Fig. 3. f3:**
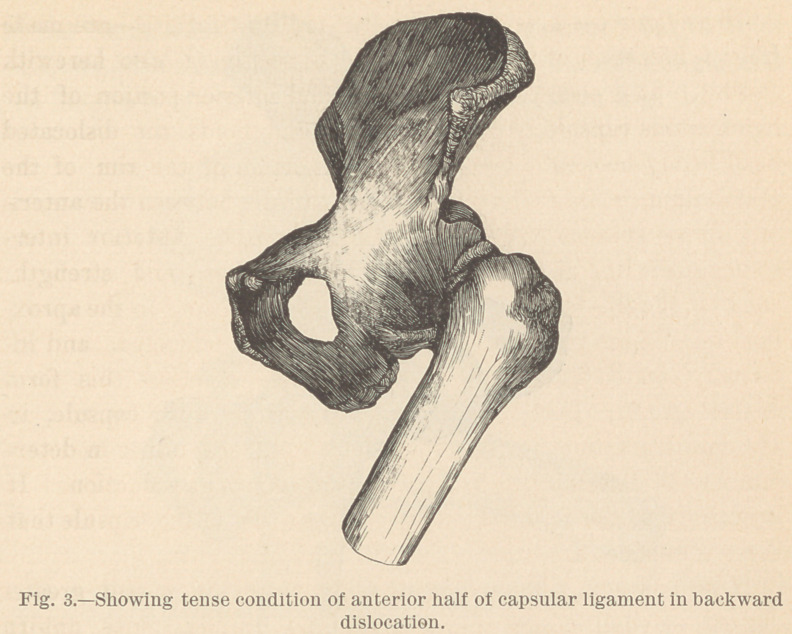


**Fig. 4. f4:**
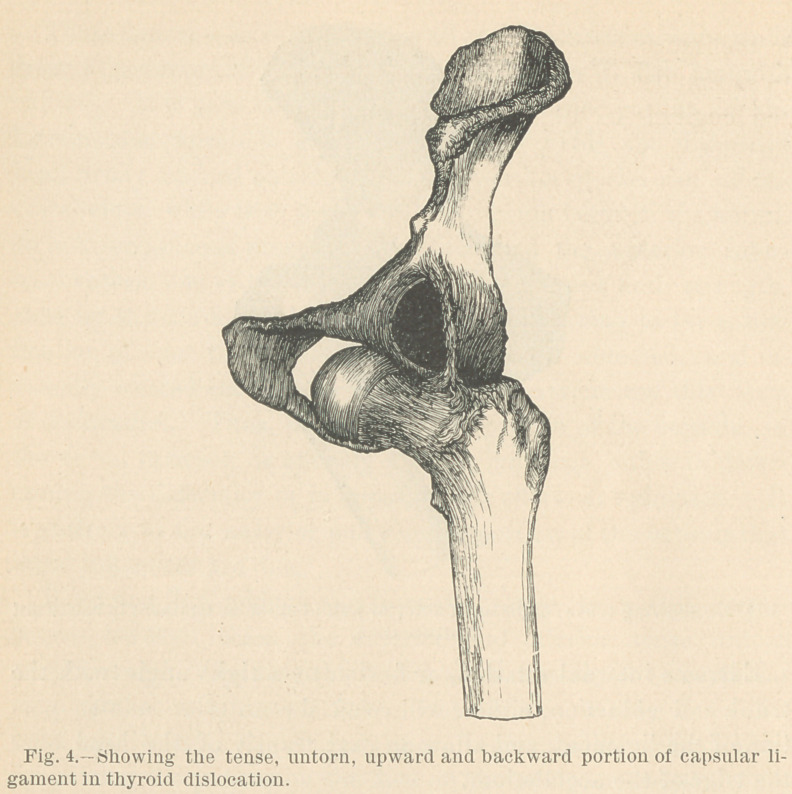


**Fig. 5. f5:**
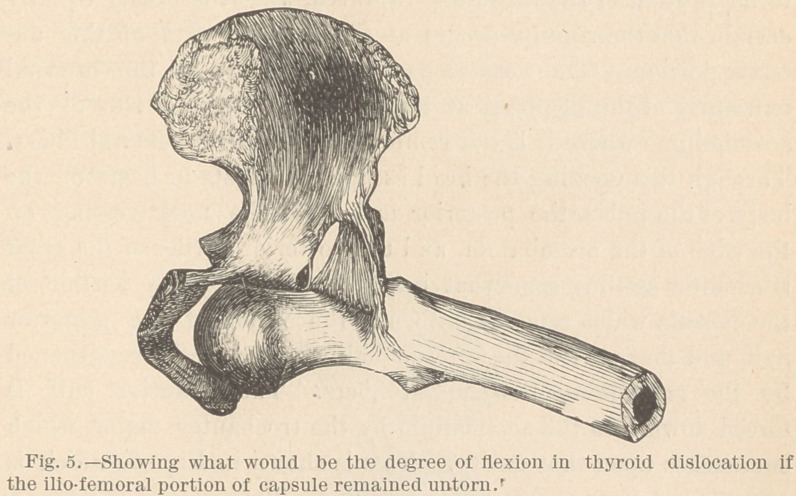


**Fig. 6. f6:**
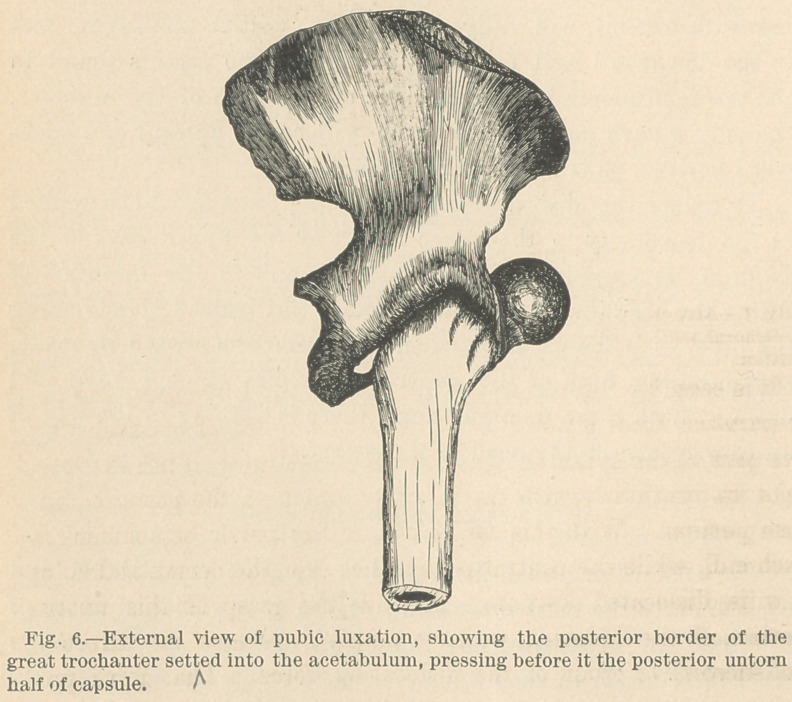


**Fig. 7. f7:**
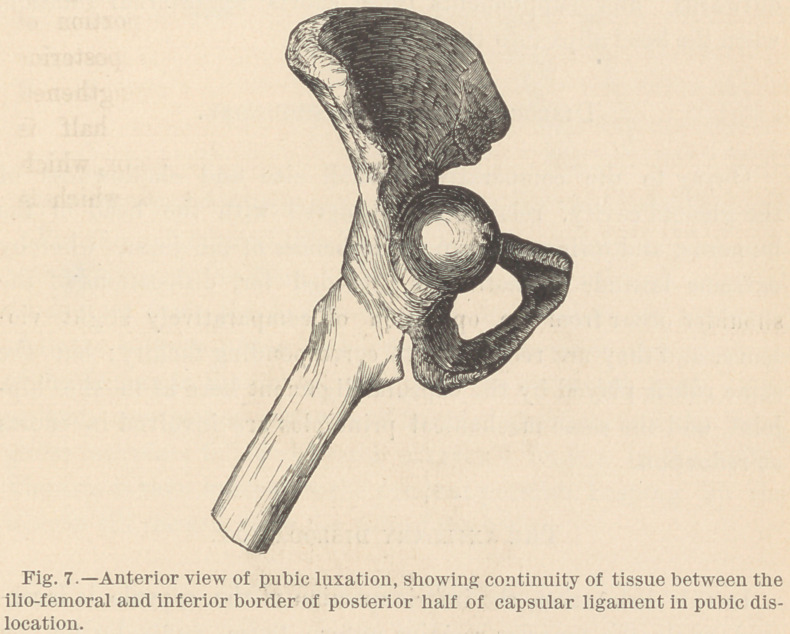


**Fig. 8. f8:**
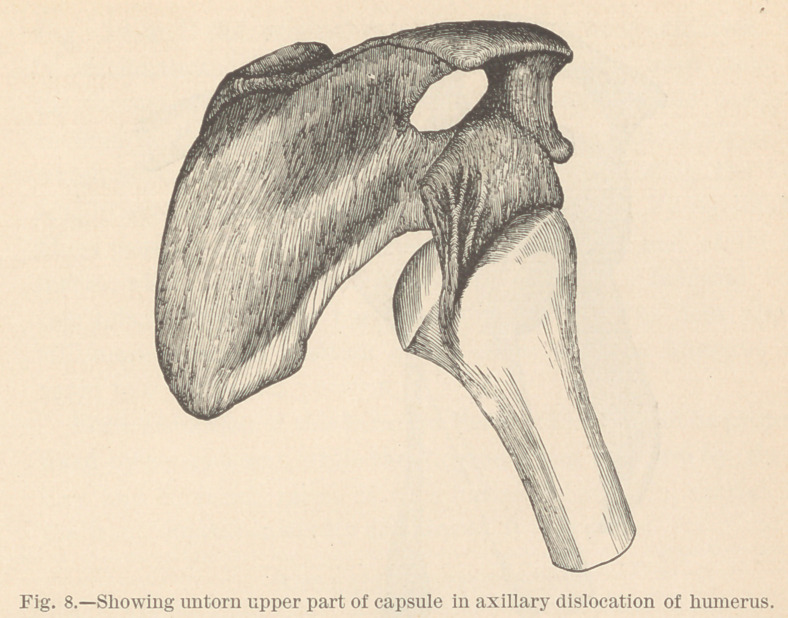


**Fig. 9. f9:**
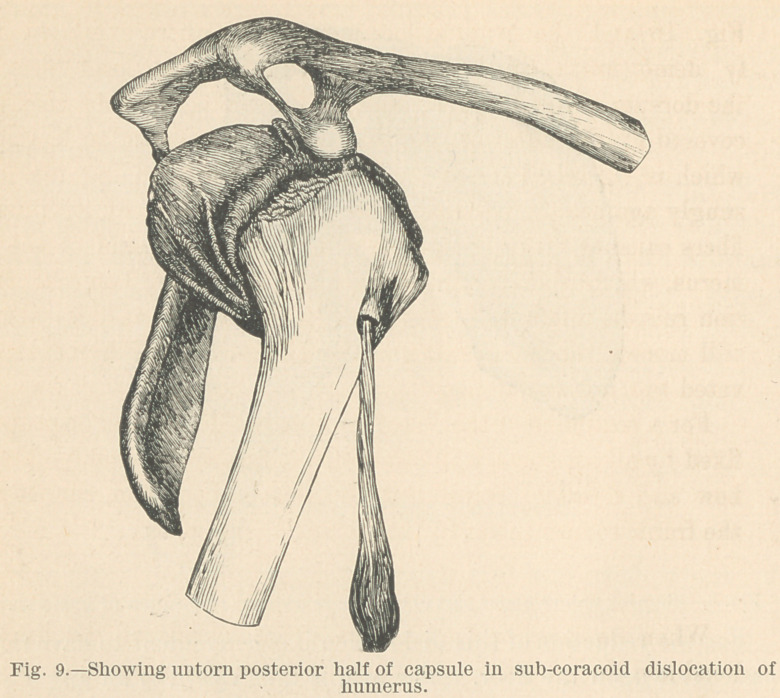


**Fig. 10. f10:**