# Errata

**Published:** 1884-05

**Authors:** 


ERRATA.—In the article on Reduction of Dislocations, by Prof. Gunn, published in this number, the following corrections should be made:

Page 451, twentieth line from top, for seven read three.

Page 462, in description of Fig. 6, for setted read settled.

Page 466, in description of Fig. 10, for posterior read anterior.

